# Healthcare Cybersecurity Ethical Concerns during the COVID-19 Global Pandemic: A Rapid Review

**DOI:** 10.3390/healthcare11222983

**Published:** 2023-11-18

**Authors:** Cristian Lieneck, Matthew McLauchlan, Sean Phillips

**Affiliations:** 1 School of Health Administration, Texas State University, San Marcos, TX 78666, USA; 2 College of Business, Concordia University-Texas, Austin, TX 78726, USA; jmatthew.mclauchlan@ctx.edu (M.M.); sean.phillips@ctx.edu (S.P.)

**Keywords:** cybersecurity, COVID-19, pandemic, ethics, ethical principles, healthcare

## Abstract

Background and objectives: Healthcare organizations have endured significant challenges and relied upon telehealth and related technological advances during the COVID-19 pandemic to allow for the sustainment of care. The purpose of this study was to systematically identify healthcare cybersecurity ethical concerns experienced during the pandemic to assist with the sustainability of the delivery of care going forward. Methods: This study was guided by Preferred Reporting Items for Systematic Reviews and Meta-Analysis protocols for systematic reviews and focused on cybersecurity in healthcare organizations that published articles during the COVID-19 pandemic (March 2020 through October 2022). The articles were accessed using the EBSCOhost and Pub-Med (which queries MEDLINE) platforms, through which the Academic Search Complete, MEDLINE Complete, and Complementary Index databases were accessed. Follow-on supplementary topic modeling allowed for the additional application of ethical principles to the review findings. Results: Among the 22 articles that met the inclusion criteria, three ethical concerns were identified by the rapid review: smart and medical technology concerns (73% of occurrences), at-risk population cybersecurity (55% of occurrences), and legal challenges in data protection (73% of occurrences). The research team also conducted a latent Dirichlet allocation (LDA) analysis, identifying three topics from the review corpus: robotic and biomedical/clinical care outcomes, diagnostic applications, and public health data usage. These were then mapped to primary ethical healthcare principles. Conclusions: The sustainment of healthcare technology interoperability and related telehealth initiatives involves the ongoing assessment of cybersecurity threats and adequate knowledge of related ethical stakeholder concerns to promote ongoing care delivery.

## 1. Introduction

### 1.1. Background

The novel coronavirus disease of 2019 (COVID-19) was declared a worldwide global pandemic by the World Health Organization in March 2020. As a result, healthcare organizations across the globe have adapted to public health physical distancing and related measures to prevent the spread of the virus. The provision of medical care during a pandemic was therefore challenged due to the presence of multiple variables, including inventory (personal protection equipment) shortages, limited availability of medical providers, and even healthcare organization infrastructure issues [1]. Prior research demonstrates that telehealth and the use of healthcare technological advances have been relied upon to help sustain the provision of care, coordination of care, and throughput of patients [2,3,4,5]. However, the increased use of technology and telehealth practices comes with security concerns, specifically regarding cybersecurity [6].

Unfortunately, privacy concerns and data breaches in healthcare organizations flourished during the COVID-19 pandemic. By the end of 2021, it was reported that over 40 million patient records had been compromised by hacking groups and the use of ransomware, bringing down hospital networks for weeks or even months at a time [7]. In the United States alone, it has been cited that over 600 healthcare organization data breaches occurred in 2021 during the pandemic [8], potentially compromising over 22.6 million patients [9]. As a result, the need for heightened cybersecurity efforts and precautions are required, especially during global environmental challenges such as COVID-19.

Cybersecurity is the initiative taken by organizations to protect their technological assets (networks, devices, and especially organizational data) from unauthorized access and/or illegal (criminal) use [10]. In the healthcare industry, the protection of private health information is often regulated by governmental agencies and stringently enforced. Specific to healthcare, cybersecurity has been heavily researched, with best practices, modern trends, and potential solutions having been investigated [11,12]. Systematic literature reviews, from prior to the COVID-19 pandemic and beyond, have been published; however, such systematic reviews [12,13] do not investigate, assess, or analyze how such cybersecurity threats (and related organizational initiatives in response to such threats) implicate healthcare stakeholders’ ethical principles. The purpose of this study was to address the question of what cybersecurity ethical concerns were experienced during the COVID-19 pandemic, with the intention of furthering the sustainability of the delivery of care.

### 1.2. Rationale

The research team was further intrigued by the lack of research that specifically cited healthcare ethical principles when addressing unethical cyber-behaviors and data security risks during the pandemic. Prior studies surrounding cybersecurity during COVID-19 certainly detail violations of ethical principles, but often fail to directly map (or code) potentially unethical behaviors to such healthcare ethical principles. For instance, Mierzwa et al. [14] have addressed the need for attention to be paid to ethics across different disciplines and even cites the Center for Internet Security (CIS) rules of Cyber Ethics, providing a proposed outline (checklist) to help reduce cyberattacks. Yet, primary healthcare ethical principles are not mapped to unethical behaviors. Research by Williams et al. [15] has suggested a number of factors that make individuals, organizations, and even employers more vulnerable to cyberattacks in healthcare, but did not focus directly on prior attacks during COVID-19 and the ethical principles potentially being violated. Finally, Middaugh [16] has addressed specific cyberattacks during the pandemic, while providing four risk categories associated with hospital ransomware attacks, nurse manager responsibilities, and what to do to prevent such attacks. As a result, the research team wanted to specifically focus on healthcare industry cyberattacks identified in the literature and map such behaviors to healthcare ethical principles. To our knowledge, this is the only systematic literature review that has focused on codifying cybersecurity attacks and related issues during the pandemic to healthcare ethical principles using supplementary topic modeling.

Following the increased use of telehealth to maintain operations, the sustainment of such practices is believed to have continued in many sectors of the healthcare industry. As the infection rates of COVID-19 continue to drop and symptoms are better able to be controlled by vaccines and boosters, routine care is expected to continue to return, potentially reaching pre-pandemic levels eventually. But despite the feats that healthcare technology enabled medical providers and their healthcare organizations to accomplish during the height of the pandemic surges and now, the threat of cybersecurity remains a risk. As digital attacks continued to occur and even became more common in various industries during the pandemic, the assessment of cybersecurity ethical implications for healthcare stakeholders is warranted to identify both challenges and concerns. The ability to address such issues will assist with the sustainability of healthcare technology and telehealth practices as COVID-19 variants continue to arise.

### 1.3. Objectives

The objective of this review was to determine the underlying healthcare industry cybersecurity concerns and/or threats to the sustainment of care during the COVID-19 global pandemic. An additional goal was to understand what ethical principles are potentially affected by cyber threats and related healthcare technology challenges. The rationale behind this review and the follow-on topic modeling construct validation was to provide insight into ethical concerns related to cybersecurity challenges in healthcare systems to allow for insight into initiatives to address issues and sustain the healthcare technology advances that were made during the pandemic.

## 2. Methods

### 2.1. Eligibility/Inclusion Criteria

Articles included in the review were required to focus on healthcare cybersecurity ethical initiatives and/or challenges experienced during the COVID-19 pandemic. Only original peer-reviewed academic journals were utilized in the search initiative. The review team decided upon an aggressive publication date search criteria for use in article identification (1 March 2020 to 1 October 2022) to ensure that findings were specifically related to cybersecurity ethical concerns during the pandemic. The search was conducted by the research team from 10 to 14 October 2022.

### 2.2. Information Sources

Three research databases were utilized in the review: Academic Search Complete, MEDLINE Complete, and Complementary Index. The Texas State University’s library website, the Ebson B. Stephens Company (EBSCO host), and the PubMed (which queries MEDLINE) platform provided full-text access to the research articles. The three databases were chosen for the review based upon the total number of identified articles in the initial database search (yielding the highest number of potential articles identified). Furthermore, the research team decided early in the search process to not include any terminology or search criteria related to ethics/ethical concerns in order to prevent the unnecessary elimination of potential research articles related to cybersecurity during the pandemic.

### 2.3. Search Details

**Figure 1:**
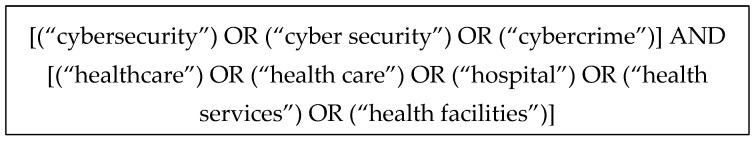
The research database search string and Boolean search operators that yielded the highest frequency of results in the search.

The search string developed by the research team involved multiple iterations of database queries to identify the highest initial search results (number of articles identified), while still meeting the inclusive terminology criteria (Figure 1).

The National Library of Medicine’s Medical Subject Headings (MeSH) controlled thesaurus was utilized to index research articles found through PubMed (MEDLINE) and was utilized by the research team to identify key words for the research database query search string to focus specifically on two themes: cybersecurity and the healthcare industry. The research team identified the MeSH (exploded) key words for the review. Multiple database searches were conducted to identify appropriate Boolean operators to identify the search thread with the highest initial review sample. Because the study was limited to COVID-19-related articles only, additional snowballing searches proved ineffective, because this primarily led to articles published prior to COVID-19, which therefore did not meet the inclusion criteria of this study.

### 2.4. Initial Study Selection

The review was guided by Preferred Reporting Items for Systematic Reviews and Meta-Analysis (PRISMA) [17]. The entire research team participated in the initial database search. To be as inclusive as possible, the research team did not require that articles fulfil the full-text EBSCO host optional article exclusion option (yielding the maximum number of results). Once the initial sample of articles was identified, the research team then collated all the articles in full-text format and saved them to both an MS Teams site and also a reference management software program for citation purposes.

The research team met multiple times via telephone and webinar to identify and review all articles from the initial search selection criteria. Construct identification and discussion was conducted using an MS Excel spreadsheet, which also captured individual comments and thoughts surrounding each article’s inclusion in the review. The review team did not experience any disagreements regarding article inclusion/exclusion decisions for the study, including with regard to the underlying theme (construct) identification and categorization.

### 2.5. Latent Dirichlet Allocation (LDA) Method

The research team further analyzed the review articles using an additional topic modeling analysis method in an attempt to further validate the three constructs (themes), while also working to map these themes to healthcare ethical principles as appropriate. This process involved a computer-aided text analysis (CATA) technique (topic modeling) to review the healthcare cybersecurity articles identified in the review process. For the purposes of this study, latent Dirichlet allocation was the preferred method for topic modeling and follow-on analysis [18].

LDA assumes a document (each identified article) to be a collection of multiple topics, and that all of the words in the manuscript can be used to learn the topics included in each document. A probabilistic inference approach is used in LDA to identify latent topics from observed words and/or word clusters. As such, LDA does not require any pre-establishment of criteria or keywords for coding beforehand [19,20,21].

The research team utilized the Google Code Archive graphical user interface topic modeling tool (TopicModelingTook.wiki), which utilizes the MALLET toolkit for analyses. The LDA analysis was applied all 22 review articles’ abstracts, which were cleaned when copied/pasted into a single .txt file by removing (deleting) any hard returns, paragraph symbols, and article subtitles.

## 3. Results

### 3.1. Study Selection and Exclusion Process

**Figure 2:**
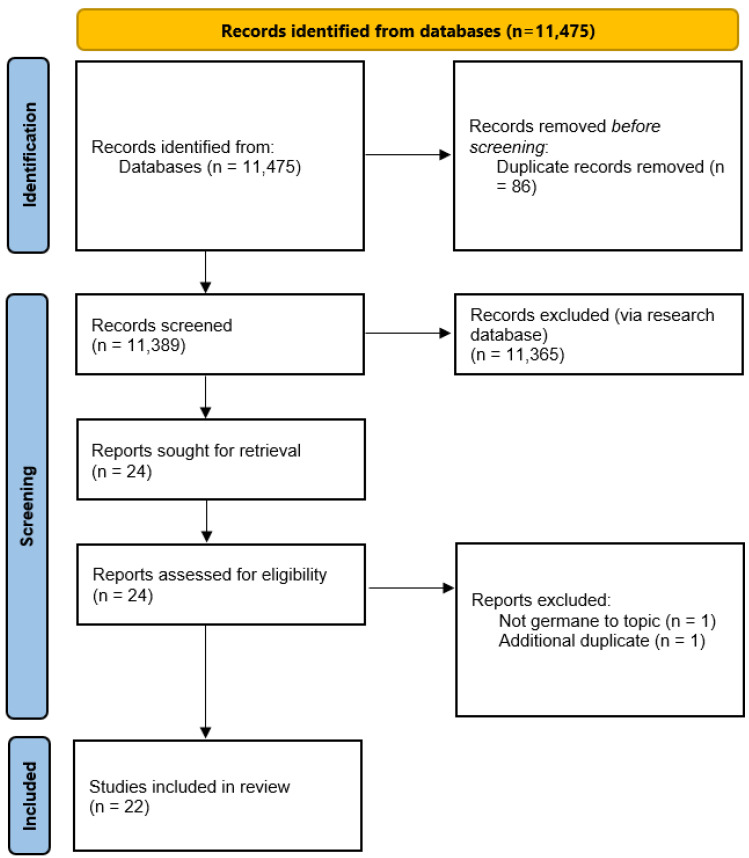
A diagram detailing the preferred reporting items for systematic reviews and meta-analyses (PRISMA) that demonstrates the study selection process.

Figure 2 demonstrates the study selection and the exclusion process, which initially identified 11,475 articles from the research databases. From articles published within the search date criteria, 86 duplicates were identified and initially removed from the search using the EBSCO host website feature. Of the remaining articles, additional EBSCO host exclusion options were utilized to identify articles specifically related to the research topic. Subsequent filtering processes eliminated 11,365 additional articles based upon the research team’s required criteria:

(a) English-only manuscripts;
(b) Peer-reviewed, and/or academic journals only.

A full-text review was conducted of the remaining 24 articles by the three-member reviewer team. Each article was reviewed by at least two research team members, with several articles being reviewed by multiple researchers (Table 1).

Upon the completion of the full-text review process, an additional two articles were removed due to them being not germane to the research topic (1) or identified as an additional article duplicate (1). The ‘not germane to topic’ article excluded did not meet the search topic criteria but was somehow identified by the initial library research database search engine. Upon the completion of the full-text review, the research team decided upon the inclusion of the remaining 22 identified articles (n = 22).

### 3.2. Study Characteristics

The underlying themes (constructs) were identified by the research team upon studying the 22 identified articles concerning cybersecurity during the COVID-19 pandemic and its ethical implications. Cybersecurity concerns included healthcare organizations’ initiatives to address potential implications, and some researchers simply citing concerns during pandemic-related public health and health system challenges (Table 2). Due to the limited number of articles in the review, an individual quality appraisal/evaluation of the 22 studies occurred during the full-text review, conducted by the research team, but no further studies were eliminated from the review.

### 3.3. Data Analysis/Results

**Figure 3:**
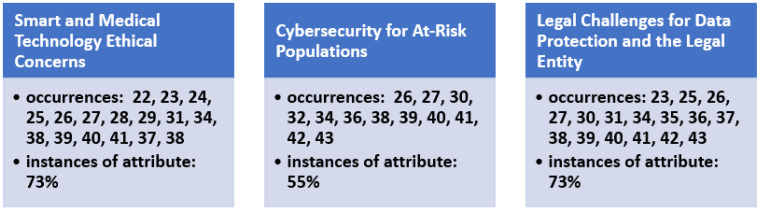
The occurrence of cybersecurity ethical constructs underlying themes identified as observed in the literature.

The results of the review process demonstrated three main cybersecurity ethical themes in the literature. The research team conducted this review as part of a graduate-level healthcare management course in healthcare ethics, therefore focusing specifically on ethical challenges related to cybersecurity in the healthcare industry during COVID-19, as identified in the review articles (Figure 3).

### 3.4. Supplementary Analysis—Topic Modeling

#### 3.4.1. Latent Dirichlet Allocation Results

The initial analysis yielded results with topics including both “health” or “health care” and “cybersecurity.” This suggests that the review team’s efforts on the initial article selection and use of appropriate exploded MeSH vocabulary was successful. However, these initial results did not specifically meet the research team’s topic analysis purpose of identifying and/or mapping potential healthcare cybersecurity concerns during the COVID-19 pandemic onto ethical principles. For example, the research team wanted to identify more-detailed topics under the “smart and medical technology ethical concerns” theme. Therefore, the analysis was re-run after deleting those words. The raw LDA outputs show the most common words and their weights. For example, the analysis was re-run after deleting these words and the keywords in the first topic included, among others, robotic clinical (0.8%), patient (0.6%), and biomedical outcomes (0.4%). Because a topic is a mixture of words, when a word has high probability of being in a topic, all the documents having the word will be more associated with the topic as well. After examining the abstracts and these keywords, the research team identified the topic proportions, as listed in Table 3.

Robotic and biomedical/clinical care and outcomes can be categorized under smart and medical technology ethical concerns. Diagnostic applications are related to cybersecurity for at-risk populations, while public health data usage matches legal challenges for data protection. Consequently, the subjects uncovered through LDA analysis align with the constructs found in the team’s systematic review (from manual examination), indicating that LDA analysis serves as a reliable method for recognizing the themes of articles in literature review research. These findings also support the notion that machine learning methods can aid researchers in confirming the selection of samples and in detecting textual themes.

#### 3.4.2. Application of LDA Topic Modeling Results to Review Findings

**Figure 4:**
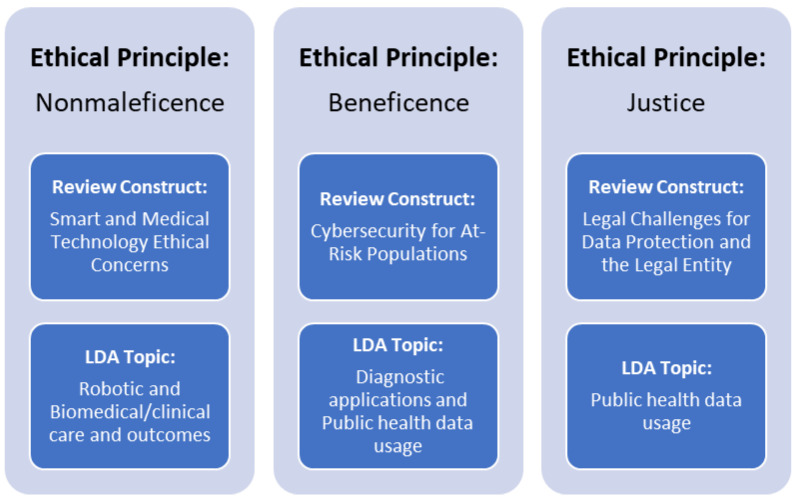
Relationships of ethical principles, as identified in the review and follow-on LDA analysis.

Using this approach, the research team labeled the top three topics from the LDA outputs. Multiple iterations (200) were conducted with the stop words removed, and these three topics were able to be mapped to healthcare industry ethical principles by the research team based on the LDA theme categorization, as well as abstract content surrounding underlying ethical principles related to each manuscript. These findings and their relationship with the initial constructs identified via systematic review are summarized in Figure 4.

As a result, the topics identified via the LDA analysis are consistent with the systematic review findings (constructs identified via the manual review). This suggests that the LDA analysis is an effective tool in identifying the articles’ topics in literature review studies and provides evidence that machine learning techniques may support the validation of the sample selection (review articles) and identify textual and latent topics.

## 4. Discussion

### 4.1. Summary of Evidence and Ethical Principles

The concept of ethical considerations in healthcare has been extensively explored and integrated across diverse global healthcare systems. In particular, autonomy, nonmaleficence, beneficence, and justice stand as the central pillars guiding contemporary healthcare provision [44]. Each of these principles, with its distinct objectives, holds sway over all facets of healthcare delivery, encompassing domains like healthcare technology, telehealth, and telemedicine. Ensuring the lasting viability of such technologies demands a continuous evaluation of ethical quandaries and the repercussions faced by stakeholders within the industry. While the review team identified numerous isolated instances reflecting various healthcare ethical principles, the analysis highlighted three fundamental principles that consistently emerged in both the rapid review and LDA topic modeling analyses.

The research team pinpointed nonmaleficence (the principle of “first, do no harm”) [44] as an ethical cornerstone impacted by the discerned structure of the review, encompassing ethical dilemmas in smart and medical technologies. This was also a key LDA topic, represented by robotic and biomedical/clinical care results. This principle revolves around the idea that care delivery should not involve any genuine harm, should bring about advantageous outcomes for the patient, should exclude any erroneous actions, and ultimately, that the positive results must outweigh any negative aspects.

Beneficence (making choices with an overarching intent to promote goodness) [44] emerged as an ethical foundation flagged by the research team, identified by review framework as concerning cybersecurity considerations for vulnerable populations and by the LDA discourse as encompassing diagnostic applications and the utilization of public health data. It is imperative for healthcare practitioners to consistently center their attention on every individual patient, taking into account their unique needs, desires, and overall care experience. This selfless dedication on the part of medical providers is essential in every patient interaction, irrespective of the patient’s attributes or demographics (such as religious affiliation, personal convictions, and even individual preferences).

Justice (ensuring equitable treatment and care) [44] stands as another ethical principle spotlighted in this study, affected by cybersecurity apprehensions within the context of the legal intricacies surrounding data protection in the review framework and by the LDA topic that concerns the utilization of public health data. The utilization of healthcare data, even in aggregate form, should consistently incorporate measures to uphold patient privacy. Furthermore, the equitable application of health-related research—including the reporting and utilization of data sets during the COVID-19 pandemic—demands the implementation of justice at the level of individual patient care.

### 4.2. Ethics in Cybersecurity, Specifically in Smart and Medical Technology

The review team initially identified a primary theme in the literature surrounding ethical implications for healthcare stakeholders utilizing smart and medical technology devices as part of their treatment. As a result, three sub-themes for this construct were identified and are discussed in Section 4.2.1, Section 4.2.2 and Section 4.2.3.

#### 4.2.1. Testing Cyberattacks

Ensuring the security of collected data and the types of tests employed in patient care plays a pivotal role in safeguarding against cyberattacks and upholding an organization’s commitment to ethical principles. Frequently, assessments of cyber vulnerability can identify system weaknesses without compromising personal information [27]. The literature suggests that the execution of simulated scenarios be tested, wherein a controlled environment is created to probe potential system vulnerabilities through hacking simulations [31]. In such instances, real patient data would not be applicable or used for these tests. Nevertheless, given the potential exposure to multifarious cyber threats like ransomware at different levels, comprehensive research and testing involving users and infrastructure are necessary, all while safeguarding patient data from wearables or other electronic record systems [43].

#### 4.2.2. Patient-Facing Robotics and Electronics

The healthcare market is continually introducing novel medical equipment, often encompassing advanced robotic surgical tools and electronic systems that have the potential for interoperability. It is imperative that these interoperable devices are not only well-supported but also securely operated, regardless of the volume of individual patient data stored on any given device. Communities, such as the elderly and LGBTQ+ patients, seek assurance that emerging technologies are entirely safeguarded against data breaches [36]. Progressions in innovations like brain–computer interfaces (BCI) prompt discussions about the ethical boundaries of brain information and the efficacy of its security measures [39]. As health data becomes increasingly digitized each year, the importance of such safeguards becomes more pronounced.

Similar scenarios arise during digital sign-ins for office appointments and the utilization of digital medical recording devices by healthcare providers [41]. In virtually every sphere, medical equipment, including robots, is now employed to access our medical data. Cybersecurity must ensure the protection of all such information, necessitating the integration of ethical considerations [35]. Ethics will play a pivotal role in bolstering the sustainable development of IT infrastructure, carrying a heightened responsibility encompassing care ethics, biomedical standards, technical norms, and cybersecurity [38].

#### 4.2.3. Artificial Intelligence (AI)

A primary focal point involves the utilization of AI to bolster cybersecurity capabilities and fortify data protection, alongside its application in conducting extensive big data analyses and shaping treatment protocols for medical practitioners. It is imperative that AI remains impervious to undue influence and consistently upholds the ethical codes governing medical professionals [40]. Robust cybersecurity protocols are essential to safeguard AI algorithms and associated code within healthcare technologies, thus averting data corruption and potential implications for patient care. The incorporation of AI capabilities can further enhance strategies in cybersecurity, catering to both public and private medical domains [31]. The establishment of ethical frameworks encompassing autonomy, consent, and beneficence necessitates a comprehensive approach, such as the UN SHAPES project’s ethical guidelines, designed to cater to the diverse layers of stakeholders involved [32].

### 4.3. Demographics of Users, Specifically At-Risk Populations

The progression of information and communication technology (ICT) has yielded advantages across various domains, encompassing e-health services, e-transport, smartphones, and the sector of virtual assistants/household robots [33]. Among these, a focal point has been the elderly population, garnering particular attention within the European Union (EU). Regrettably, while they have reaped benefits, it has also exposed them to potential abuse, including cyberbullying targeting the silver generation [34]. This is concerning, as the integration of technology into their homes exposes the elderly and LGBTQ+ individuals, who are already more vulnerable segments of society. The adoption and utilization of technology raise numerous security concerns for these susceptible population groups [35,36].

Distinguishing between the concepts of “safety” and “security,” research delineates safety’s focus on circumstances that could harm living beings, while security pertains to the intricate IT infrastructure and data management [35]. With the integration of healthcare technology into everyday life, as seen in the emergence of “smart cities” that collect information extensively, the user demographics expand, encompassing both tech-savvy individuals and at-risk populations. Ethical approaches exhibit a global diversity, resulting in a wide array of ethical programming toolkits [23]. This diversity underscores the increasing significance of established norms [23,24], potentially leading to a global standardization for safeguarding medical information within integrated information systems.

### 4.4. Legal Issues Surrounding Requirements for Data Protection and Legal Entity

Advanced healthcare systems empowered by high technology hold the promise of enhancing healthcare outcomes and opportunities. The integration of AI, care robots, and telemedicine within healthcare, coupled with seamless computer network integration, facilitates the seamless provision of health-related services. For instance, AI’s capacity for continuous decision making, intricate task execution, and workload reduction positions it as a transformative tool for legal practice [30,31]. Similarly, care robots, employed in surgical procedures, assistance, and rehabilitation, underline the technological advancement while also necessitating sound security protocols [29,32]. Telemedicine amplifies patient care and advice, creating a demand for interactivity, accessibility, and health informatics, thereby raising legal considerations associated with these transactions [32].

The migration of healthcare systems toward high-tech applications exposes them to cyber threats that can disrupt healthcare operations and services. Often, the negative impact of potential cyberattacks outweighs the positive effects. Concerns are notably raised by the public regarding cyberattacks on digital health due to the lack of robust jurisdiction to govern digital data sharing [42]. Analyzing the trends, it is clear that stricter cybersecurity regulations are on the rise. An encompassing legal understanding of AI’s role is pivotal for an effective adaptation process [30], leading to a growing need for solutions. The European Union has initiated methods for standardization and policy formulation for AI, aiming to establish reliability through accreditation and a legal framework [40].

The ascent of AI necessitates an evolving comprehension of regulations, triggering inquiries for further development and prompting necessary adjustments to legal structures [36]. Amid the potential for advancement, uncertainties linger regarding the legality of cybersecurity, encompassing issues of privacy and access, leading to legal concerns [25,39]. Similarly, just as care robots present challenges in terms of cyberattacks, they underscore the demand for heightened security, protection, and safety due to the nature of their hardware and data exchange. As the current legal documents on network security, regulations, and cybersecurity were not tailored specifically for care robots, stakeholders need to address this gap through focused cybersecurity legal frameworks [29,40].

An alternative avenue involves leveraging legal ethics within cybersecurity ethics to prevent damage and illicit operations [38]. However, this approach is still in its nascent stages. Even the mere sharing of digital information can lead to dissemination issues. The risks of misinformation via social media platforms were laid bare during the pandemic, highlighting the need for precise legal measures to counter harmful information. The pandemic indirectly led to questioning the existing legal systems’ capacity to handle challenges to democracy and health [22]. Consequently, ICT-driven alternatives that optimize data usage, cybersecurity, transparency, and robust applications within municipal regulations offer a clear direction [23]. This underscores the necessity for compliance and stringent rules to ensure public health protection. Often, managing healthcare provider data internally, whether involving AI, care robots, or social media, exposes vulnerabilities to cyberattacks such as through ransomware, underscoring the cybersecurity community’s role in prevention due to gaps in current legal provisions [24]. Through educational initiatives, gamified learning techniques for cybersecurity progression can potentially overcome obstacles and stand in contrast to the non-technical aspects of legality [26]. The potential for effective strategic resolutions lies ahead in the future.

## 5. Conclusions

The protection of healthcare data and the systems which coordinate the provision of care will continue to drive cybersecurity efforts going forward. The sustainability of ethical principles related to cybersecurity efforts must continue to be considered in order to assess all stakeholder values, expectations, and perceptions of care and related outcomes. Access to valuable medical information is vital to the delivery of care, as is the speed and convenience of access measures. Healthcare leaders will continue to be responsible for the security of all health information, while upholding the highest ethical standards.

This review identified three occurrences of ethical constructs (underlying themes) related to cybersecurity efforts conducted during the COVID-19 global pandemic. Supplementary topic modeling at a basic level allowed the research team to map these constructs onto three healthcare principles: nonmaleficence, beneficence, and justice. Cybersecurity testing, the use of healthcare robotics (that utilize patient information), artificial intelligence, the security of at-risk patient populations, and organizational legal issues are all considerable concerns for healthcare leaders as cybersecurity efforts continue.

This review underscored the critical ethical concerns surrounding healthcare cybersecurity, highlighting the delicate balance between technological advancement and the protection of sensitive patient data. The limited number of articles identified signals a clear need for expanded research in this field, emphasizing the importance of incorporating a wider range of databases to ensure a comprehensive literature capture. Future research should also address the rapid evolution of the cybersecurity industry, with a focus on developing adaptable and forward-thinking ethical frameworks that can keep pace with the swift advancements in technology. Such practices will help safeguard patient information and help to enhance trust in healthcare systems globally, ensuring that cybersecurity measures are both effective and ethically sound.

Prior research and publications surrounding cybersecurity during the COVID-19 pandemic have identified the potential vulnerabilities of healthcare organizations, provided precautional steps to help prevent information breaches and cybersecurity threats, and discussed the ramifications of such events occurring [45,46,47]. This research identified the underlying healthcare ethical constructs within the current peer-reviewed literature surrounding cybersecurity efforts during the pandemic, while also mapping the themes to related healthcare organizational actions/activities. Ongoing research will be required to ensure any future concerns surrounding cybersecurity efforts during ongoing and/or future COVID-19 viral spread limits the potential contravention of ethical principles for all healthcare stakeholders.

## Figures and Tables

**Table 1:** Reviewer assignment of the initial database search findings (full-article review).

Article Assignment	Reviewer 1	Reviewer 2	Reviewer 3
1–5	X	X	X
5–10		X	X
10–15	X		X
15–22	X		X

**Table 2:** Summary of findings (n = 22).

Article No.	Author Name(s)	Article Title	Publication	Identified Ethical Cybersecurity Concerns
[22]	Barrett, T.	Negotiating Democracy and Deontology in a Pandemic	Voices in Bioethics	Review of the ethical requirements and consequences of voting during the COVID-19 pandemic (2020), reviewing deontological and consequentialism. Reviews the drive to provide different avenue to vote in secure methods. Utilizes a deontological approach to secure voting activity and the consequentialism of not providing secured voting controls or safety to those susceptible to COVID-19. Conclusion: the abnormally of voter habits and the increased use of alternative voting methods created complications for the democratic process.
[23]	Belli, Luca Maganhoto Doned, and Danilo Cesar	Municipal Data Governance: An Analysis of Brazilian and European Practices	Revista de Direito da Cidade	Assesses the concept of a “smart city”, including the collection and utilization of data for the benefit of planning and optimization of urban centers. Addresses the access and operationalization of the data collected potentially for unethical uses. Synthesis of reports and review of Rio de Janeiro and Barcelona projects and related municipal laws and best practices. Highlights the ethical values applied to ICTs, including the development of an acceptable ethics toolkit.
[24]	Botes, Marietjie; Lenzini, Gabriele	When Cryptographic Ransomware Poses Cyber Threats: Ethical Challenges and Proposed Safeguards for Cybersecurity Researchers	2022 IEEE European Symposium on Security and Privacy Workshops	Intending to understand the harmful software impact on systems facing inadequate lawful practices. Evaluates cyberspace protection and the protocol that necessitates proper cybersecurity using dialogue and research analysis. Point out that the continuous effort to participate in the study’s advancement is advantageous to overcoming the obstacles of internetworking.
[25]	Butt, Sidra Azmat; Draheim, Dirk	Ethical Challenges of ICT for the Silver Economy	2021 Eighth International Conference on eDemocracy and eGovernment (ICEDEG)	Used information and communication technologies (ICT) to address the needs of the changing demographics of an aging population in the European Union (EU) and Baltic Sea Region (BSR) and its potential benefits and risks. Synthesized a secondary review of demographic ICT-collected data vs. data from primary sources or computed findings. The technology proposals present benefits to an aging population. The risk of compromising integrity, privacy, affordability, and access, along with the potential of dehumanization and unhealthy overdependence need to be considered within the structured roll out of ICT in the EU and BSR.
[26]	DeBello, Joan E.; Schmeelk, Suzanna; Dragos, Denise M.; Troja, Erald; Truong, Laura M.	Teaching effective Cybersecurity through escape the classroom paradigm.	2022 IEEE Global Engineering Education Conference (EDUCON)	Intended to educate on cyberspace protection and its patterns. Using a research explanation, the strategy and difficulty of cyber safety during academic lessons was analyzed. The outcomes reveal that in game-based learning, there was a tendency toward stumbling upon obstacles more so than with the traditional form.
[27]	DeCusatis, Casimer; Peko, Patrick; Irving, Jordan; Teache, Maxwell; Laibach, Christopher; Hodge, Jason	A Framework for Open-Source Intelligence Penetration Testing of Virtual Health Care Systems	2022 IEEE 12th Annual Computing and Communication Workshop and Conference (CCWC)	The research looked to attempt different ways of penetrating remote healthcare services from various points in the system. Using ethical approaches, the most vulnerable aspects of the system will be tested, such as social engineering attacks, network-based attacks, and website attacks.
[28]	Formosa, Paul; Wilson, Michael; Richards, Deborah	A principle list framework for cybersecurity ethics	Computers and Security	This research looked to tackle ethical issues that arise within the space of cybersecurity practices and technology. In being able to address the subject, the paper proposes that an ethical framework be used by introducing the principle list ethical framework.
[29]	Giansanti, Daniele; Gulino, Rosario Alfio	The Cybersecurity and the Care Robots: A Viewpoint on the Open Problems and the Perspectives	Healthcare	The aim was to explore the task of the autonomous machine providing support in computer networking systems, figure out the issues, the moral inference, and the processes. The method incorporated various research databases and manuscripts to uncover the abnormality in cyberspace. The results show that cyberattacks exist, affecting operations, software, and network connection, and additional research involving machines and health needs to be conducted to improve the insight.
[30]	Golbin, Ilana; Rao, Anand S.; Hadjarian, Ali; Krittman, Daniel	Responsible AI: A Primer for the Legal Community	2020 IEEE International Conference on Big Data (Big Data)	The purpose was to comprehend the negative effect of the robotized judgment of artificial intelligence (AI) and the demand for higher-quality regulations. Assessed various prospectuses and instructions as the feedback mechanism for the growing demands regarding procedures of AI applied in the system. The result proved no significant change in the outcome, but the increase in the legitimate means of authority, practice, and position of AI adoption is valuable for the public.
[31]	He, Ying; Ni, Kun; Luo, Cunjin	Attacking Pathways of Health Information System (HIS)	2021 Computing in Cardiology (CinC)	This paper looked at the recent development in further cyberattacks on health information systems (HIS) over the years. By simulating an environment with opensource healthcare information, the group was able to hack and analyze ways to improve security efforts.
[32]	Kaplan, Bonnie	Revisiting Health Information Technology Ethical, Legal, and Social Issues and Evaluation: Telehealth/Telemedicine and COVID-19	International journal of medical informatics	The study sought to find and recap problems in digital health systems with only online access or telecare, to improve the understanding of the recent pandemic and examine the effect further. The method used a research review with procedures and a chart to document the problem of principles and public health in a computerized system. Indicated highly apparent demands for interactivity, network protection, detail accessibility, and health informatics.
[33]	Kim, J.	The Need for Stricter Control of social media by the US Government During the COVID-19 Epidemic	Voices in Bioethics	Intended to inform the dissemination of inaccurate message delivery during the pandemic crises that affects the public and suggests a mandate for more strong directives. Evaluated the online media channels impending other reliable sources through research and reports. The results showed that some media cooperation increases to reinforce positive health facts, but more action and abiding by the law are critical to securing people’s wellness.
[34]	Lane, Rhiannon	Expanding boundaries in psychiatry: uncertainty in the context of diagnosis-seeking and negotiation.	Sociology of Health & Illness	Addressed the impact of the self-diagnosis of psychological disorders or the negotiation of diagnosis with patients and providers. Review of sample of 26 pre- and post-diagnostic mental health settings through three CMHTs and one-opinion psychiatry. Conclusion: human nature has an impact on seeking diagnosis or discounting psychiatric diagnosis along with uncertainty regarding the ability to diagnose beyond patient narratives. Therefore, the ontological uncertainty is not communicated to the patient. the use of dimensional models to emphasize severity and symptoms may be more appropriate.
[35]	Monoscalco, Lisa; Simeoni, Rossella; Maccioni, Giovanni; Giansanti, Daniele	Information Security in Medical Robotics: A Survey on the Level of Training, Awareness and Use of the Physiotherapist	Healthcare	Review of the impact of interconnected medical devices (IMB) and cybersecurity in healthcare across the spectrum of users and providers (incl. builders). Used an electronic questionnaire to investigate the acceptance and consensus of physiotherapists (n = 316) across a cross section of topics: demographics, robotic/cybersecurity, training, self-assessment, proposal and collection of cyber-risk. Confirmation that robotics has the potential for use in the healthcare (physiotherapist) domain. Therefore, due to the potential physical and psychological implications and potential PHI, cybersecurity has become important issue to face.
[36]	Poulsen, Adam; Fosch-Villaronga, Eduard; Burmeister, Oliver K.	Cybersecurity, value sensing robots for LGBTIQ+ elderly, and the need for revised codes of conduct.	Australia’s J. Inf. Syst.	This article looks at the developing relationship between healthcare professionals and the security of using technology and information systems. This was achieved by looking at social robots among LGBTQ+ elderly groups. The group felt at odds with different aspects of using the technology offered within their homes. Being elderly LGBTQ+, they were already a more vulnerable group in society. The tech. brought up many security concerns for them.
[37]	Puşcã, Corneliu Andy	Legal Aspects on the Implementation of Artificial Intelligence.	EAI Endorsed Transactions on Creative Technologies	Reviewing the legal ramifications of AI and robotics, specifically the classification and application of moral and ethics of human interaction with AI and sentient programs. Review of legal doctrine and the application of moral, ethical, and philosophical arguments on the status of ePersons (similar to incorporation) and the potential social contract inclusion (taxation/ability to contract) of AI and robots. Conclusion: with the advent of AI and robotic development, the legal and regulatory framework is inadequate to address these constructs. Efforts are required from legislators to address the situation before proliferation occurs.
[38]	Rajamäki, Jyri; Hämäläinen, Heikki	Ethics of Cybersecurity and Biomedical Ethics: Case SHAPES.	Information & Security: An International Journal	Discussion of the UN SHAPES project as it relates to the health and wellbeing service guidelines and tools for developers. A white paper detailing the value and framework for the cybersecurity of the SHAPES project developers related to health and wellbeing. Specifically aligned with the ICT project in EU and BSR states. Results/conclusion: ethics are crucial in supporting sustainable development with IT infrastructure, and a higher burden exists for ethics of care, biomedical, technical, and cybersecurity.
[39]	Sample, Matthew; Sattler, Sebastian; Blain-Moraes, Stefanie; Rodríguez-Arias, David; Racine, Eric	Do Publics Share Experts’ Concerns about Brain–Computer Interfaces? A Trinational Survey on the Ethics of Neural Technology.	Science, Technology, & Human Values	This paper looked at the relationship between the overall ethics of using brain–computer interface technology. Data was taken from Germany, Canada, and Spain for a report on concerns about ethics behind BCIs.
[40]	Sharkov, George; Todorova, Christina; Varbanov, Pavel	Strategies, Policies, and Standards in the EU Towards a Roadmap for Robust and Trustworthy AI Certification.	Information & Security: An International Journal	The objective was to identify the direction of artificial intelligence (AI) practice and accreditation for appropriately executing it and its ethical code of conduct. The method assessed the rules, moral values, and the applied AI with the obstacles and efforts through research review and analysis. The study revealed that the need to utilize and adhere to the rules of AI outweighs any further inventiveness and builds integrity in high-tech systems.
[41]	Tully, Jeffrey; Coravos, Andrea; Doerr, Megan; Dameff, Christian	Connected Medical Technology and Cybersecurity Informed Consent: A New Paradigm	Journal of Medical Internet Research	As technology continues to grow in the world of healthcare, so do the concerns of cybersecurity. This paper looked to address the different ways cybersecurity is involved with patients and certain levels of consent. The paper looked at the improvements that have been needed over the last few years, the use of software patches, and the overall possible inconsistencies in keeping software updated and secure. Toward forming a better process for creating levels of consent for both patient and clinical use of technology systems in healthcare.
[42]	Vilaza, Giovanna Nunes; Coyle, David; Bardram, Jakob Eyvind	Public Attitudes to Digital Health Research Repositories: Cross-sectional International Survey.	Journal of Medical Internet Research	The study explored how people’s perceptions affect computerized health studies to allow for adaptation and progression. The study collected participants’ feedback data by utilizing website questionnaires in a prevalence study and statical analysis. Demonstrated that knowledge or experience about health is more accountable for the impact on people.
[43]	Wilson, Richard; Iftimie, Ion	Emerging ransomware threats: An anticipatory ethical analysis	2021 IEEE International Symposium on Technology and Society (ISTAS)	This paper looked at future versions of multilevel extortion ransomware and the possible ways to combat it. Policy on preventative measures will hopefully be developed from the research.

**Table 3:** Latent Dirichlet allocation (LDA) supplementary analysis output.

Topic	Proportion (%)
Robotic and biomedical/clinical care and outcomes	40%
Diagnostic applications	30%
Public health data usage	30%

## Data Availability

Not applicable.

## References

[1] Lieneck C., Betancourt J., Daemen C., Eich R., Monty E., Petty M.J. (2021). Provision of Palliative Care during the COVID-19 Pandemic: A Systematic Review of Ambulatory Care Organizations in the United States. Medicina.

[2] Lieneck C., Ramamonjiarivelo Z., Cox J., Dominguez J., Gersbach K., Heredia E., Khan A. (2021). Patient Throughput Initiatives in Ambulatory Care Organizations during the COVID-19 Pandemic: A Systematic Review. Healthcare.

[3] Lieneck C., Weaver E., Maryon T. (2021). Outpatient Telehealth Implementation in the United States during the COVID-19 Global Pandemic: A Systematic Review. Medicina.

[4] Lieneck C., Herzog B., Krips R. (2021). Analysis of Facilitators and Barriers to the Delivery of Routine Care during the COVID-19 Global Pandemic: A Systematic Review. Healthcare.

[5] Lieneck C., Garvey J., Collins C., Graham D., Loving C., Pearson R. (2020). Rapid Telehealth Implementation during the COVID-19 Global Pandemic: A Rapid Review. Healthcare.

[6] Lieneck C., Furlong E., Morrison E.E. (2019). Technological Advances in Health Care: Blessing or Ethics Nightmare?. Health Care Ethics: Critical Issues for the 21st Century.

[7] Jercich K. (2021). The Biggest Healthcare Data Breaches of 2021. Healthcare IT News. https://www.healthcareitnews.com/news/biggest-healthcare-data-breaches-2021.

[8] U.S. Department of Health and Human Services (2023). Breach Portal: Notice to the Secretary of HHS Breach of Unsecured Protected Health Information. https://ocrportal.hhs.gov/ocr/breach/breach_report.jsf.

[9] Davis J. 10 Biggest Healthcare Data Breaches of 2021 Impact over 22.6 M Patients. SC Media. https://www.scmagazine.com/feature/breach/10-biggest-healthcare-data-breaches-of-2021-impact-over-22-6m-patients.

[10] Cybersecurity & Infrastructure Security Agency (2009). Security Tip (ST-4-001): What Is Cybersecurity?. https://www.cisa.gov/uscert/ncas/tips/ST04-001.

[11] Conn J. Federal Task Force Takes on Healthcare Cybersecurity. Modern Healthcare. http://www.modernhealthcare.com/article/20160416/MAGAZINE/304169890.

[12] Kruse C.S., Frederick B., Jacobson T., Monticone D.K. (2017). Cybersecurity in Healthcare: A Systematic Review of Modern Threats and Trends. Technol. Health Care.

[13] Luna R., Rhine E., Myhra M., Sullivan R., Kruse C.S. (2016). Cyber threats to health information systems: A systematic review. Technol. Health Care.

[14] Mierzwa S., RamaRao S., Jackson T. Global Ethical and Societal Issues and Considerations with Cybersecurity in Digital Health: A rapid review. Proceedings of the Northeast Decision Sciences Institute Annual Conference.

[15] Williams C.M., Chaturvedi R., Chakravarthy K. (2020). Cybersecurity Risks in a Pandemic. J. Med. Internet Res..

[16] Middaugh D.J. (2021). Cybersecurity Attacks during a Pandemic: It Is Not Just IT’s Job!. MEDSURG Nurs..

[17] (2020). PRISMA Checklist. Transparent Reporting of Systematic Reviews and Meta-Analyses. http://www.prisma-statement.org/.

[18] Asmussen C.B., Møller C. (2019). Smart literature review: A practical topic modelling approach to exploratory literature review. J. Big Data.

[19] Holder A.K., Karim K., Lin J., Woods M. (2013). A content analysis of the comment letters to the FASB and IASB: Accounting for contingencies. Adv. Account. Inc. Adv. Int. Account..

[20] Boritz J.E., Carnaghan C., Alencar P.S. (2014). Business modeling to improve auditor risk assessment: An investigation of alternative representations. J. Inf. Syst..

[21] Chiu V., Liu Q., Vasarhelyi M.A. (2014). The development and intellectual structure of continuous auditing research. J. Account. Lit..

[22] Barrett T. (2020). Negotiating Democracy and Deontology in a Pandemic. Voices Bioeth..

[23] Belli L., Doneda D. (2020). Municipal Data Governance: An Analysis of Brazilian and European Practices. Rev. Direito Cid..

[24] Botes M., Lenzini G. When Cryptographic Ransomware Poses Cyber Threats: Ethical Challenges and Proposed Safeguards for Cybersecurity Researchers. Proceedings of the 2022 IEEE European Symposium on Security and Privacy Workshops (EuroS&PW).

[25] Butt S.A., Draheim D. Ethical Challenges of ICT for the Silver Economy. Proceedings of the 2021 Eighth International Conference on eDemocracy & eGovernment (ICEDEG).

[26] DeBello J.E., Schmeelk S., Dragos D.M., Troja E., Truong L.M. Teaching Effective Cybersecurity Through Escape the Classroom Paradigm. Proceedings of the 2022 IEEE Global Engineering Education Conference (EDUCON).

[27] DeCusatis C., Peko P., Irving J., Teache M., Laibach C., Hodge J. A Framework for Open Source Intelligence Penetration Testing of Virtual Health Care Systems. Proceedings of the 2022 IEEE 12th Annual Computing and Communication Workshop and Conference (CCWC).

[28] Formosa P., Wilson M., Richards D. (2021). A Principlist Framework for Cybersecurity Ethics. Comput. Secur..

[29] Giansanti D., Gulino R.A. (2021). The Cybersecurity and the Care Robots: A Viewpoint on the Open Problems and the Perspectives. Healthcare.

[30] Golbin I., Rao A.S., Hadjarian A., Krittman D. Responsible AI: A Primer for the Legal Community. Proceedings of the 2020 IEEE International Conference on Big Data (Big Data) 2020.

[31] He Y., Ni K., Luo C. Attacking Pathways of Health Information System (HIS). Proceedings of the 2021 Computing in Cardiology (CinC).

[32] Kaplan B. (2020). Revisiting Health Information Technology Ethical, Legal, and Social Issues and Evaluation: Telehealth/Telemedicine and COVID-19. Int. J. Med. Inform..

[33] Kim J. (2020). The Need for Stricter Control of Social Media by the US Government During the COVID-19 Epidemic. Voices Bioeth..

[34] Lane R. (2020). Expanding Boundaries in Psychiatry: Uncertainty in the Context of Diagnosis-Seeking and Negotiation. Sociol. Health Illn..

[35] Monoscalco L., Simeoni R., Maccioni G., Giansanti D. (2022). Information Security in Medical Robotics: A Survey on the Level of Training, Awareness and Use of the Physiotherapist. Healthcare.

[36] Poulsen A., Fosch-Villaronga E., Burmeister O.K. (2020). Cybersecurity, Value Sensing Robots for LGBTIQ+ Elderly, and the Need for Revised Codes of Conduct. Australas. J. Inf. Syst..

[37] Puşcã C.A. (2020). Legal Aspects on the Implementation of Artificial Intelligence. EAI Endorsed Trans. Creat. Technol..

[38] Rajamäki J., Hämäläinen H. (2021). Ethics of Cybersecurity and Biomedical Ethics: Case SHAPES. Inf. Secur. Int. J..

[39] Sample M., Sattler S., Blain-Moraes S., Rodríguez-Arias D., Racine E. (2020). Do Publics Share Experts’ Concerns about Brain–Computer Interfaces? A Trinational Survey on the Ethics of Neural Technology. Sci. Technol. Hum. Values.

[40] Sharkov G., Todorova C., Varbanov P. (2021). Strategies, Policies, and Standards in the EU Towards a Roadmap for Robust and Trustworthy AI Certification. Inf. Secur. Int. J..

[41] Tully J., Coravos A., Doerr M., Dameff C. (2020). Connected Medical Technology and Cybersecurity Informed Consent: A New Paradigm. J. Med. Internet Res..

[42] Vilaza G.N., Coyle D., Bardram J.E. (2021). Public Attitudes to Digital Health Research Repositories: Cross-sectional International Survey. J. Med. Internet Res..

[43] Wilson R., Iftimie I. Emerging Ransomeware Threats: An Anticipatory Ethical Analysis. Proceedings of the 2021 IEEE International Symposium on Technology and Society (ISTAS).

[44] Furlong E., Morrison E.E. (2019). Health Care Ethics: Critical Issues for the 21st Century.

[45] McKeon J. (2023). Biggest Healthcare Data Breaches Reported This Year, So Far. Health IT Security. https://healthitsecurity.com/features/biggest-healthcare-data-breaches-reported-this-year-so-far.

[46] Southwick R. (2023). Here Are the 10 Biggest Health Data Breaches in the First Half of 2023. Chief Healthcare Executive. https://www.chiefhealthcareexecutive.com/view/here-are-the-10-biggest-health-data-breaches-in-the-first-half-of-2023.

[47] Vogel S. Scale of Healthcare Cyber Attacks Increase as Criminals Change Tactics, Report Finds. Healthcare Dive. https://www.healthcaredive.com/news/cyber-attacks-healthcare-scale-increase-critical-insights/691478/.

